# Motor Imagery Classification Using Mu and Beta Rhythms of EEG with Strong Uncorrelating Transform Based Complex Common Spatial Patterns

**DOI:** 10.1155/2016/1489692

**Published:** 2016-10-03

**Authors:** Youngjoo Kim, Jiwoo Ryu, Ko Keun Kim, Clive C. Took, Danilo P. Mandic, Cheolsoo Park

**Affiliations:** ^1^Department of Computer Engineering, Kwangwoon University, 20 Gwangun Rd, Nowon-gu, Seoul 01897, Republic of Korea; ^2^LG, 38 Baumoe-ro, Seocho-gu, Seoul 137724, Republic of Korea; ^3^Department of Computing, University of Surrey, Guildford, Surrey GU27XH, UK; ^4^Department of Electrical and Electronic Engineering, Imperial College London, South Kensington Campus, London SW72AZ, UK

## Abstract

Recent studies have demonstrated the disassociation between the mu and beta rhythms of electroencephalogram (EEG) during motor imagery tasks. The proposed algorithm in this paper uses a fully data-driven multivariate empirical mode decomposition (MEMD) in order to obtain the mu and beta rhythms from the nonlinear EEG signals. Then, the strong uncorrelating transform complex common spatial patterns (SUTCCSP) algorithm is applied to the rhythms so that the complex data, constructed with the mu and beta rhythms, becomes uncorrelated and its pseudocovariance provides supplementary power difference information between the two rhythms. The extracted features using SUTCCSP that maximize the interclass variances are classified using various classification algorithms for the separation of the left- and right-hand motor imagery EEG acquired from the Physionet database. This paper shows that the supplementary information of the power difference between mu and beta rhythms obtained using SUTCCSP provides an important feature for the classification of the left- and right-hand motor imagery tasks. In addition, MEMD is proved to be a preferred preprocessing method for the nonlinear and nonstationary EEG signals compared to the conventional IIR filtering. Finally, the random forest classifier yielded a high performance for the classification of the motor imagery tasks.

## 1. Introduction

The development of the brain computer interface (BCI) system allows one to control and communicate with the surrounding environment [1]. This includes applications ranging from education and entertainment to environmental control and communication through a noninvasive measurement of electroencephalogram (EEG) [2–4]. In particular, measuring EEG during different motor imagery tasks (e.g., left- and right-hand motor imagery) is a widely used paradigm to implement a BCI system. The EEG data acquired during these motor imagery tasks is then classified in order to control the BCI system.

The cornerstone to generate control signals that will facilitate the classification of different mental tasks is to extract the appropriate features from the obtained EEG data. A number of different methods were used to perform feature extraction for various signals [5, 6], such as principal component analysis (PCA), which was used to classify additional forward tasks and relaxation [7] as well as left- and right-hand motor imagery tasks [8]. Independent component analysis (ICA) was also applied to motor imagery tasks (e.g., left- and right-hand [9] or finger lifting tasks [10]), which produced a high classification accuracy. Common spatial patterns (CSP) algorithm is another widely used feature extraction method, which is based on the fact that such neural activities are spatially distributed on the cortex areas [11, 12]. Recently, a complex version of the CSP algorithm has been proposed to analyze two different EEG signals simultaneously in a complex form, which provides features containing the power sum information of the real and imaginary parts. The complex CSP algorithm was also combined with the strong uncorrelating transform (SUT) in order to cater for general complex signals considering the noncircularity (noncircular signals are not invariant to rotations, which may have power difference between real and imaginary parts of the complex form) of the data and maximizing the variance-ratio between two motor imagery tasks [13].

In addition, the power sum of the mu and beta rhythms gained from the complex version of CSP is closely related to the event-related phenomena, indicating the changes of the frequency components in the EEG data. Due to the synchronization in neuronal activities, the phenomena of a decrease (event-related desynchronization, ERD) [14, 15] or an increase (event-related synchronization, ERS) [16] in power of frequency components were found [17]. The brain lateralization of ERD [18] of the EEG activity during motor imagery tasks of the left- and right-hand is also well known [13, 19].

There have been further attempts to analyze EEG signals by investigating the different frequency band components of EEG separately, such as the mu (8–13 Hz) and beta (13–25 Hz) rhythms. This is based on the fact that the beta rhythm has distinct topographies and responses to the limb movements, compared to the mu rhythm, and thus the mu and beta rhythms should be individually considered [20, 21]. Brinkman et al. showed that the oscillatory power of the mu rhythm in the sensorimotor cortex ipsilateral to the tasks increased, while that of the beta rhythm in the contralateral sensorimotor cortex decreased simultaneously [22]. However, many of the previous studies on motor imagery responses analyzed the data considering all frequency components as a whole, which ignores the difference between the mu and beta rhythms [11, 13, 23]. In order to utilize the different information from the mu and beta rhythms for improved performance of motor imagery classification, we propose the application of SUTCCSP by constructing a complex formed data of these two rhythms.

Furthermore, considering the multichannel, nonlinear, and nonstationary property of EEG signals, recent studies have proved that the empirical mode decomposition (EMD) based algorithm is more effective than the conventional Fourier analysis in preprocessing physiological signals including motor imagery EEG signals [23–26]. In addition to the preprocessing method, various nonlinear classifiers have been applied to the classification of motor imagery tasks during the last decade [18]. The most commonly used classification method used in this area of the BCI research is the linear discriminant analysis (LDA) [27]. However, recent studies suggest that nonlinear classifiers are more practical by taking into account nonlinear relationships between data and the robustness against noise and outliers [28, 29]. Recent studies show that the well known machine learning based nonlinear classifier, random forest (RF) produces a high classification rate in the application of motor imagery classification [28, 30].

In this paper, we propose to classify the motor imagery EEG signals by analyzing the mu and beta rhythms separately in a complex form of data using SUTCCSP. EEG signals from the Physionet database [31, 32] are preprocessed using MEMD in order to decompose the signals into mu and beta rhythms. The two distinct signals are then utilized to produce a complex data, which is composed of mu and beta rhythms in its real and imaginary terms for the analysis of SUTCCSP. Taking into account the functional disassociation of mu and beta rhythms, the extracted features using this method contain valuable information of the power difference between these two distinct rhythms. In addition, random forest (RF) is used in order to classify the left- and right-hand motor imagery tasks. As a result, the SUTCCSP algorithm results in a higher classification accuracy (80.05%) compared to the conventional CSP algorithm (78.04%) that does not account for the difference between the two distinct rhythms.

## 2. Methods

### 2.1. Motor Imagery EEG Datasets

The proposed algorithms were applied to the EEG data from the Physiobank Motor/Mental Imagery (MMI) database [31–33]. The database consists of a total of 109 subjects who performed the left- and right-hand motor imagery tasks. Each subject performed a total average of 46.62 ± 0.96 trials for the left- and right-hand motor imagery tasks. The average numbers of trials were 23.62 ± 0.61 and 23.00 ± 0.62 for the left- and right-hand motor imagery data, respectively. The EEG data was sampled at 160 Hz for all subjects yielding 640 samples for each single trial. However, in this study, we excluded the data of 4 subjects including S088, S092, S100, S104, since these subjects had damaged recordings (S088, S092, and S100) and too little samples (S104) in their left- and right-hand motor imagery datasets [34]. Therefore, a total of 105 pieces of subject data out of 109 were used for the experiment.

**Figure 1:**
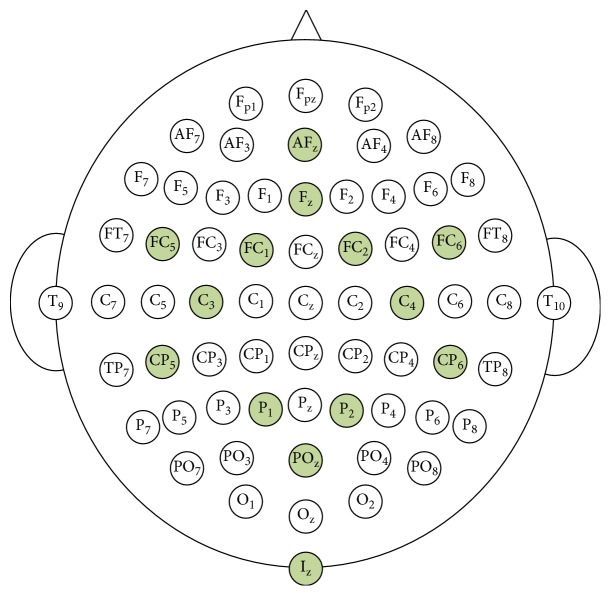
Channel selection. EEG montage of the selected 14 channels from the 64 channels provided from the Physionet database.

Out of the 64 channels of EEG data recorded with the 10-10 system, 14 channels were chosen as shown in Figure 1 for the feasible implementation of BCI with small number of channels [35]. The channels were selected so that it could cover all the regions of the scalp, including the frontal, central, parietal, and occipital lobe. Then, signals were decomposed using the MEMD in order to extract the mu and beta rhythms. After the preprocessing procedure, SUTCCSP was applied to complex formed data consisting of mu and beta rhythms, in order to extract the features for the classification of the left- and right-hand motor imagery tasks.

### 2.2. Multivariate Empirical Mode Decomposition (MEMD)

Multivariate empirical mode decomposition provides an accurate data-driven time-frequency analysis for multichannel, nonlinear, and nonstationary signals, and thus MEMD was proved to be more effective in preprocessing the motor imagery EEG signals in terms of the baseline filtering [23, 36]. With the help of MEMD, the multivariate signals were decomposed into a linear combination of multiple common oscillatory modes called intrinsic mode functions (IMFs) [37], and the baseline filtering was done by eliminating irrelevant IMFs. The original multivariate signal, **S**(*t*), is represented with the decomposed IMFs as follows:

(1) $$\begin{array}{c} S\left(\;t\;\right)=\overset{n}{\underset{k=1}{\sum }}i_{k}\left(\;t\;\right), \end{array}$$

where *i*_*k*_(*t*) represents *k*th IMF. When the original empirical mode decomposition (EMD) algorithm was applied to each channel of the data, the resulting IMFs of the same order from different channels did not have similar frequency characteristics (mode mixing problem) [38]. MEMD resolves this problem by using the mean envelope of the projected signals on a multidimensional projection space.

**Figure 2:**
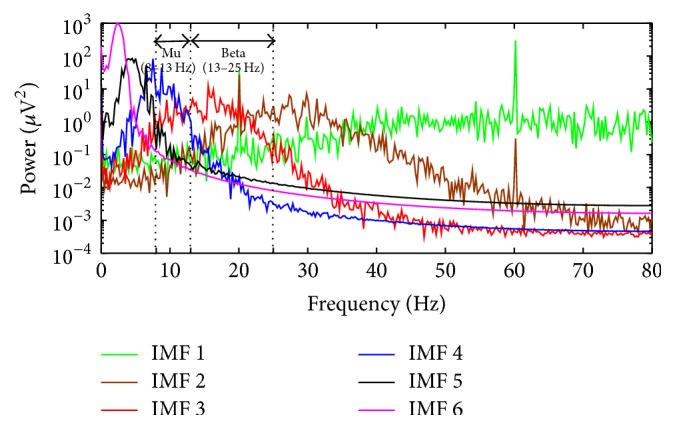
Average power spectra of all trials of 105 subjects. The power spectra show the first six IMFs, 1st IMF to the 6th IMF out of a total of 11 IMFs, which were decomposed in order using MEMD. *i*_2_(*t*) and *i*_3_(*t*) + *i*_4_(*t*) correspond to the mu (8–13 Hz) and beta rhythms (13–25 Hz), respectively. The high peak at 60 Hz, indicating the power noise, was not to be considered.

In this paper, the noise-assisted MEMD was applied to the motor imagery EEG data in order to further reduce the mode mixing problem by using an additional channel with white Gaussian noise [23]. Therefore, we used the noise-assisted MEMD to extract reliable frequency components. The IMFs that corresponded to the mu and beta rhythms were selected by investigating the power spectra of the IMFs calculated using the periodogram (Bartlett window) [39]. Bartlett window was mainly used since it was easy to implement and the aliasing problem was not as critical as the much simpler rectangular window. The averaged spectra of the 105 pieces of subject data using all trials were investigated and Figure 2 displays the averaged power spectra of the first 6 IMFs out of all 11 IMFs (from *i*_1_(*t*) to *i*_6_(*t*)). The parameters for FFT points and window size were both set to 640. As shown in Figure 2, *i*_2_(*t*), *i*_3_(*t*), and *i*_4_(*t*) cover the frequency bands of the mu and beta rhythms, and thus *i*_4_(*t*) and *i*_3_(*t*) + *i*_2_(*t*) of each trial were used as the real and imaginary parts of the constructed complex data, respectively.

### 2.3. Strong Uncorrelating Transform Complex Common Spatial Pattern

Falzon et al. first proposed the complex version of common spatial patterns in order to discriminate EEG responses to mental tasks using analytic signal-based CSP (ACSP) with Hilbert transform [40]. However, Hilbert transform could only be applied to narrowband signals, and thus empirical mode decomposition was used to produce more accurate narrowband signals compared to the Fourier analysis [41, 42].

In addition to ACSP, there have been approaches that consider the noncircularity of complex signals [13]. When a complex random variable, *z* = *z*_*μ*_ + *jz*_*β*_ is defined, covariance (**C**) and pseudocovariance (**P**) are derived as follows [43]:

(2) CEzzH=Ezμ+jzβzμ−jzβT=EzμzμT+zβzβT+jEzβzμT−zμzβT=Ezμ2+zβ2≥0,

(3) PEzzT=Ezμ+jzβzμ+jzβ=EzμzμT−EzβzβT+jEzβzμT+zμzβT=Ezμ2−zβ2+2jEzμzβ,

where *E*[·] indicates the statistical expectation operator and (·)^*H*^ and (·)^*T*^ denote the Hermitian and transpose of a vector, respectively. Equation (2) shows that the covariance contains the sum of power information of the mu and beta rhythms, whereas (3) shows that the pseudocovariance includes the power difference and the correlation information of the mu and beta rhythms. When the given data is circular, (3) is equivalent to zero, since *z*_*μ*_ and *z*_*β*_ have the same variance (*E*[*z*_*μ*_ *z*_*μ*_^*T*^] − *E*[*z*_*β*_ *z*_*β*_^*T*^] = 0) and are uncorrelated (*E*[*z*_*β*_ *z*_*μ*_^*T*^ + *z*_*μ*_ *z*_*β*_^*T*^] = 0). However, most of the biological signals are noncircular in the real world [13]. Therefore, the complex form preserves the pseudocovariance information [44] and the augmented form of complex CSP (ACCSP) that holds both the covariance and pseudocovariance information can be applied.

Then, the strong uncorrelating transform combined with ACCSP is used in order to diagonalize the covariance and pseudocovariance matrices simultaneously, assuring that the multichannel complex data can no longer be correlated [13]. The diagonalization process leaves the sum of power and the power difference information of mu and beta rhythms from the augmented covariance and pseudocovariance matrix. The steps for SUTCCSP is described below:

When given the number of channels (*N*) and sample size (*S*), **A**_*L*_ and **A**_*R*_ are both *N* × *S* matrices that hold the zero-mean data of the two classes, left-hand (*L*) and right-hand (*R*) motor imagery tasks, respectively. In this paper, obtained *i*_4_(*t*) and *i*_3_(*t*) + *i*_2_(*t*) that cover the mu and beta rhythms of the EEG data recorded during motor imagery tasks are used as the real and imagery terms shown as **A**_*L*  or  *R*_ = *i*_4_(*t*) + *j*(*i*_3_(*t*) + *i*_2_(*t*)). (original CSP algorithm uses **A**_*L*  or  *R*_ = *i*_2_(*t*) + *i*_3_(*t*) + *i*_4_(*t*).)

Then, the covariance and pseudocovariance matrices of **A**_*L*_ and **A**_*R*_ can be generated as follows:

(4) CL=cov⁡AL=EALALH,CR=cov⁡AR=EARARH,PL=pcov⁡AL=EALALT,PR=pcov⁡AR=EARART.

Then, a composite spatial covariance and pseudocovariance matrices are calculated as

(5) Cc=CL+CR=EALALH+EARARH,Pc=PL+PR=EALALT+EARART.

Using the eigen decomposition, there exists a factorization form of

(6) $$\begin{array}{c} C_{c}=U_{c}\;\Lambda_{c}\;U_{c}^{H}, \end{array}$$

so that **C**_*c*_ can be whitened by applying whitening matrix **G** = Λ_*c*_^−1/2^ **U**_*c*_^*H*^, **I** = **G** **C**_*c*_ **G** ^*H*^, and the pseudocovariance matrix is then decomposed using Takagi's factorization as follows [13]:

(7) $$\begin{array}{c} {\bar{P}}_{c}=GP_{c}G^{T}=Y\Lambda Y^{T}. \end{array}$$

The SUT transform matrix **Q** is defined as

(8) $$\begin{array}{c} Q=Y^{H}G. \end{array}$$

Therefore, SUTCCSP is a transform of the whitened factorization form of the covariance and pseudocovariance matrices. The process above allows both the covariance and pseudocovariance matrices to be diagonalized simultaneously as

(9) QCcQH=QCLQH+QCRQH=I,QPcQH=QPLQT+QPRQT=Λ.

Assuming **S**_*L*_ = **Q** **C**_*L*_ **Q** ^*H*^ and **S**_*R*_ = **Q** **C**_*R*_ **Q** ^*H*^, the SUT transform yields an estimation of the eigenvectors from the covariance matrix so that

(10) ΛL=B−1SLB,ΛR=B−1SRB.

The estimation of the eigenvectors from the pseudocovariance matrix is also obtained as follows:

(11) Q^=Λ−1/2YHG,

(12) S^L=Q^PLQ^T,

(13) Q^PcQ^T=S^L+S^R=I,

(14) B^−1S^LB^=Λ^L,B^−1S^RB^=Λ^R,

where $\hat{B}$ and $\hat{\Lambda }$ indicate the eigenvectors and eigenvalues of $\hat{S}$, respectively. The derived equations (10) and (14) lead to Λ_*L*_ + Λ_*R*_ = **I** and ${\hat{\Lambda }}_{L}+{\hat{\Lambda }}_{R}=I$, which is equivalent to Λ_*L*_ = **I** − Λ_*R*_ and ${\hat{\Lambda }}_{L}=I-{\hat{\Lambda }}_{R}$. Therefore, if the values of Λ_*L*_ and ${\hat{\Lambda }}_{L}$ are in descending order, values of Λ_*R*_ and ${\hat{\Lambda }}_{R}$ will be in the ascending order. This is the main property of the CSP algorithm, which illustrates that the variance of one class is maximized, while the variance of the other class is minimized, when applying the following covariance and pseudocovariance spatial filter:

(15) W=B−1G,W^=B^−1G^.

The final step to extract the features is to apply spatial filter **W** to given data **A** as

(16) V=WA,V^=W^A,

where **V** and $\hat{V}$ are the covariance and pseudocovariance spatial filtered data, respectively, and their power values are used as features. These obtained features are then separated using classifiers to classify the left- and right-hand motor imagery tasks. To demonstrate the advantage using the pseudocovariance information, we show the complex version of CSP (CCSP), using the similar procedure to SUTCCSP, only using the covariance information in **W**, except $\hat{W}$. Therefore, unlike SUTCCSP preserving both the power sum and difference information, CCSP only preserves the power sum information of the real and imaginary terms of the complex variable. In this way, we show how features containing the power difference information affect the classification accuracy of the motor imagery tasks.

### 2.4. Classifiers

In this study, five machine learning algorithms, including the random forest (RF), logistic model tree (LMT), model tree (MT), *k*-nearest neighbor (KNN), and logitboost (LB) [30, 45–49], were implemented using WEKA and utilized for the benchmark test of classifiers [27, 28]. These five classifiers have frequently been used to classify various motor imagery tasks [45, 50–53]. These classifiers are mainly based on bagging or boosting (random forest, logistic model tree, model tree, and logitboost) [30, 46, 47, 49]. In addition to the classifiers based on bagging and boosting, the well known *k*-nearest neighbor algorithm, which uses the neighboring distance measures of features, was also used for the comparison of the classification methods [48]. Both bagging and boosting are based on an ensemble method, using a combination of multiple learning methods to produce a better prediction. Particularly, random forest, an extended version of bagging, has been proven to be an effective classification algorithm for the classification of motor imagery tasks and emotional dimensions using EEG signals [28, 45]. This is due to the characteristics of random forest, the robustness against outliers and noise, and the useful internal estimates of the error, correlation, and variable importance [30]. Breiman introduces the definition of the random forest as follows [30]:

**Definition 1 .**

Random forest is an ensemble of tree-structured classifiers *h*(*x*, *θ*_*k*_) (*k* ∈ *ℕ*), where random vectors {*θ*_*k*_} generated at *k*th tree are independent and identically distributed and each tree votes for the most popular class (*c* ∈ {1, −1}), given input *x* from the training set.

In addition, all parameters of each classifier including the random forest were set with the default parameters of WEKA. The number of trees for random forest was set as 100 and the maximum depth of the trees was set as unlimited. For model tree, which uses a regression model for every class value [47], the minimum number of instances per leaf was set as 4. Logitboost performs an additive logistic regression [49] and the percentage of the weight mass used for base training was set as 100 with a total of 10 iterations. Logistic model tree is also based on the linear logistic regression models. However, it uses logitboost and regression functions as base learners [46] and the number of iterations for early heuristic stopping was set as 50. Finally, index *k* from *k*-nearest neighbor algorithm was set as 1.

## 3. Results

### 3.1. Classification of the Left- and Right-Hand Motor Imagery EEG Data

**Figure 3:**
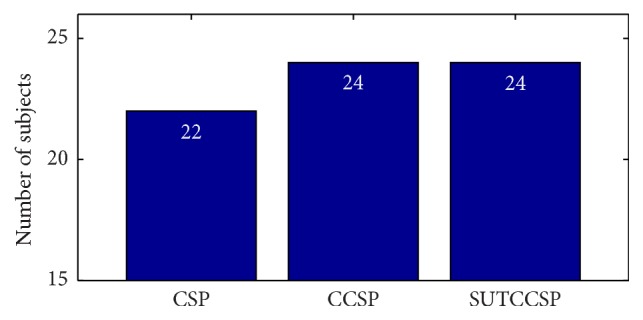
Bar chart indicating the number of significant subjects. Note that CCSP and SUTCCSP produce two more significant subjects compared to CSP.

The classification performance using the original CSP, CCSP, and SUTCCSP of significant subjects was compared using five machine learning algorithms implemented using WEKA. Machine learning algorithms include random forest, logistic model tree, model tree, *k*-nearest neighbor, and logitboost. The classification performances of all classifiers were calculated using a five-cross validation (30 iterations for different random selected training sets for each subject) and the similar analyses from [13] were applied. Specifically, significant subjects were chosen when their classification accuracies were above a certain percentage, which was set with a confidence limit of 95% (cf. [54]). The average percentage limit for 45 trials of the motor imagery tasks was approximately 64%, and thus subjects with classification rates over 64% were categorized as significant subjects. The rationale of using only significant subjects was to exclude the subjects with too low classification accuracies. Since Ahn and Jun claimed that the subjects who had performed poorly with tasks had little brain activity across the different regions of the brain or less brain network, these subjects were excluded from the evaluation [55]. The bar chart of the number of significant subjects for CSP, CCSP, and SUTCCSP is shown in Figure 3. Note that the number of significant subjects of CCSP and SUTCCSP are bigger than those of CSP.

**Figure 4:**
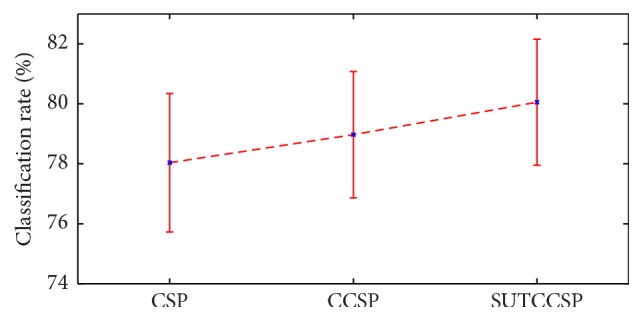
Errorbar of classification rates of the significant subjects for CSP, CCSP, and SUTCCSP. Note that SUTCCSP produces higher classification rates compared to CSP and CCSP, confirmed by Student's *t*-test in Table 3.

All subjects, who were marked significant subjects using either CSP, CCSP, or SUTCCSP, were included in the significant subject pool in order to calculate the average classification rate across the subjects. As a result, a total of 24 subjects were chosen as significant subjects, and the classification rates calculated using the five classifiers are shown in Table 1. Overall, SUTCCSP produced the highest classification rate among the CSP algorithms when classified using random forest as shown in Table 1. Additionally, error bars for these results are displayed in Figure 4, which show that SUTCCSP yields higher classification rates among the CSP algorithms. The classification accuracies for the insignificant subjects were also shown in Table 2 to compare with those for the significant subjects in Table 1. Table 2 shows that the performances of the insignificant subjects were close to 50%, which were consistent with what random chance might produce. For this, subjects yielding low performance were not appropriate for the evaluation, and, thus, the insignificant subjects were excluded in the main analysis [55].

**Figure 5:**
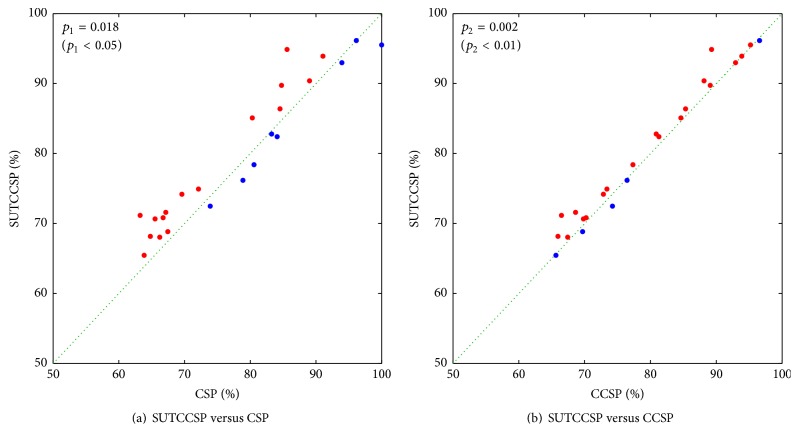
Scatterplots of the classification rates comparing SUTCCSP with CSP (a) and CCSP (b). The dots are marked red where the classification rates of SUTCCSP are larger than those of CSP or CCSP and marked blue in opposite cases. The enhanced performance using SUTCCSP is confirmed by *p* value of Student's *t*-test.

In addition, the scatterplots of the classification rates of the significant subjects are displayed in Figure 5 to compare the results of SUTCCSP with CSP (a) and CCSP (b). The diagonal lines in Figure 5 represent the cases where the classification rates of CSP or CCSP and SUTCCSP are the same. An additional study of the one-way analysis of the variance, Student's *t*-test, was conducted, where classification accuracies of SUTCCSP were compared with CSP (*p*_1_) and CCSP (*p*_2_). Note that most dots in both scatterplots lie above the line, meaning that SUTCCSP outperforms both CSP and CCSP (*p*_1_ < 0.01 and *p*_2_ < 0.005).

Table 3 shows the results of Student's *t*-test performed for the other classifiers including the random forest, which compares the classification rates of SUTCCSP with CSP (*p*_1_) and CCSP (*p*_2_). In detail, significant *p* values of random forest (*p*_1_ < 0.01 and *p*_2_ < 0.005) reassure that SUTCCSP outperforms CSP and CCSP. The other classification algorithms except the random forest gave no significance in terms of *p* values in Table 3 (*p*_1_, *p*_2_ > 0.05), and their classification rates did not vary across CSP, CCSP, and SUTCCSP in Table 1.

It is noted that the results of SUTCCSP outperformed those of CCSP, whose features include only the information of the power sum of the mu and beta rhythms. This suggests that the power difference information of them preserved by SUTCCSP can be considered as an important factor for the classification between the left- and right-hand motor imagery tasks. Therefore, results in Tables 1 and 3 prove that SUTCCSP outperforms CSP and CCSP, with random forest, a preferred classification method with CSP based feature extraction algorithms, particularly SUTCCSP.

In order to marginalize the performance difference that comes from the classifiers, the average across the performance of different classifiers was calculated for CSP (77.27%), CCSP (77.22%), and SUTCCSP (77.41%). As a result, SUTCCSP resulted in a slightly higher performance compared to the other CSP algorithms by 0.15 (%).

In addition, an additional experiment using both the CSP features and features of the power difference information of mu and beta rhythms (CSP + PD) was conducted to show that the performance improvement is coming from SUTCCSP and not solely from the random forest classifier. If our best result is yielded only from the classifying technique of the random forest classifier and not SUTCCSP, the result of CSP + PD should be comparable to that of SUTCCSP when feeding the classifier with the same amount of power difference information. However, despite using the same classifier, CSP + PD achieved a 78.63 ± 1.93 (%) classification accuracy, which is slightly higher than that of CSP but still below the best classification accuracy using SUTCCSP (80.05%) by 1.42 (%). This states that the performance improvement of the best classification accuracy is coming from the preservation of unique power difference information using SUTCCSP and does not solely come from the random forest classifier.

Additional studies comparing MEMD with IIR filtered results are presented in Tables 4 [Table tab5]–6 in order to prove that MEMD is more effective for the dataset of motor imagery EEG signals.  Table 4 shows the classification results of the preprocessed data using MEMD, which outperforms those using the 5th-order Butterworth IIR filter by approximately 1.1%, 2.9%, and 3.0% for CSP, CCSP, and SUTCCSP features, respectively. Note the largest difference between the performance of IIR and MEMD for SUTCCSP features. The classification accuracies using MEMD and the IIR filter were calculated with the average classification rate of the significant subjects among the 105 subjects.

Student's *t*-test was also utilized to compare the variances between the classification performances of MEMD and IIR prefiltered data of the significant subjects in Table 5. Results display *t*-test performed for two cases: *t*-test for the original CSP versus SUTCCSP (*p*_1_) and CCSP versus SUTCCSP (*p*_2_). Results show that the IIR filter gave no significant *p* value for *p*_1_ (>0.05) and a relatively high value for *p*_2_ (<0.05), whereas MEMD gave significantly low *p* values for both *p*_1_ (<0.01) and *p*_2_ (<0.005).

Table 6 also shows the results of Student's *t*-test performed for MEMD versus IIR filter for all 105 subjects using the different CSP algorithms. All CSP methods show significant *p* values (<0.00001), which suggest that MEMD is more competent than the IIR filter for the preprocessing of EEG. As Park et al. have shown that MEMD is effective in preprocessing motor imagery EEG signals due to the nonlinear and nonstationary characteristics of the data [23], Tables 4 [Table tab5]–6 demonstrate that MEMD is more effective than the IIR filter for this left- and right-hand motor imagery EEG dataset.

### 3.2. Spatial Pattern Topographies

**Figure 6:**
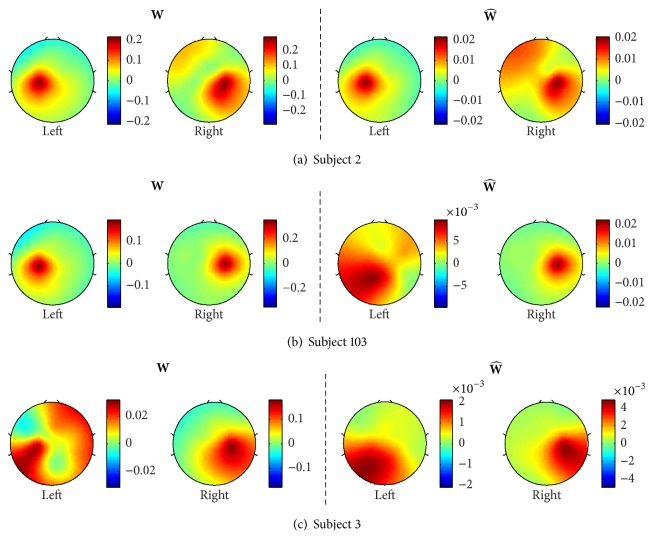
Spatial patterns of the top three subjects in descending order, who had the best classification rates: subject 34 (96.13%), 72 (95.51%), and 7 (94.89%). The left two topographies show the spatial patterns for the covariance matrices (**W**) of the left and right-hand tasks, respectively, while the right two topographies show the spatial patterns for the pseudocovariance matrices ($\hat{W}$). Note that the patterns of **W** and $\hat{W}$ are prominently different, meaning $\hat{W}$ can produce additional information to **W**.

Figure 6 illustrates the spatial pattern topographies for the top three subjects in descending order who had the best classification rates out of the 24 significant subjects: subject 34 (96.13%), 72 (95.51%), and 7 (94.89%). The left and right two topographies of Figure 6 correspond to the spatial patterns of covariance (**W**) and pseudocovariance ($\hat{W}$) matrices, respectively.

**Figure 7 fig7:**
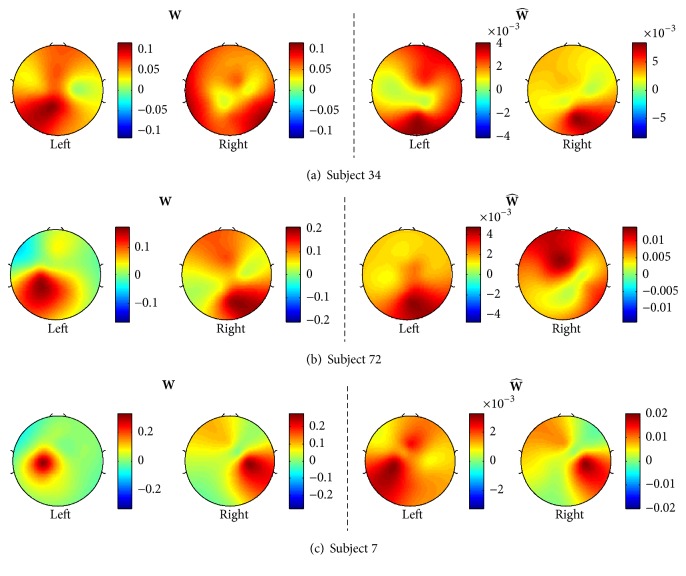
Spatial patterns of the worst three subjects in descending order, who gave the worst classification rates: subject 2 (68.18%), 103 (68.04%), and 33 (65.47%). The left two topographies show the spatial patterns for the covariance matrices (**W**) of the left- and right-hand tasks, respectively, while the right two topographies show the spatial patterns for the pseudocovariance matrices ($\hat{W}$). Note that the patterns of the left- and right-hand motor imagery are prominently different even for the subjects who had poor classification rates.

**Figure 8:**
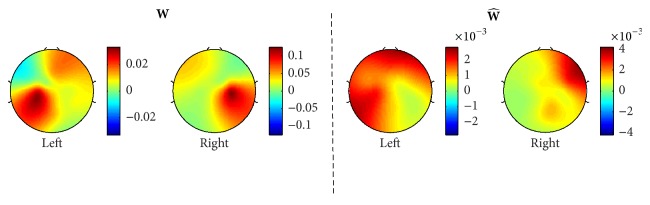
Spatial patterns of the average covariance and pseudocovariance matrices of all 24 significant subjects are shown.

Figure 7 also shows the spatial pattern topographies of the three subjects, who gave the worst classification rates: subject 2 (68.18%), 103 (68.04%), and 33 (65.47%). Overall, the typical spatial patterns during the motor imagery tasks, the synchronization on the ipsilateral hemisphere, and desynchronization on the contralateral hemisphere [19] can be noted for the covariance spatial patterns. A prominent ipsilateral power difference is also shown for the spatial patterns of the pseudocovariance of subject 33. This is also seen in Figure 8, illustrating spatial patterns of average covariance (**W**) and pseudocovariance ($\hat{W}$) matrices of all 24 significant subjects.

Furthermore, the difference between the covariance and pseudocovariance spatial patterns suggests that the pseudocovariance spatial filters provide additional information about the power difference between the mu and beta rhythms of EEG recorded during motor imagery tasks.

### 3.3. Asymmetries of the Power Difference and Sum of Mu and Beta Rhythms

**Figure 9:**
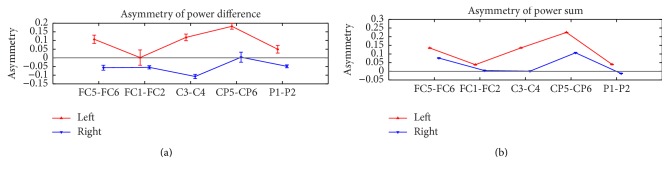
Asymmetries of the power difference and sum of symmetric channel EEG of motor imagery tasks. (a) Asymmetry of the power difference between mu and beta rhythms from the symmetric channels. (b) Asymmetry of the power sum of mu and beta rhythms from the symmetric channels. Note that the asymmetry of the power difference shows more prominent difference between the left- and right-hand motor imagery tasks with the different signs of values, compared to the asymmetry of the power sum.

Figures 6 [Fig fig7]–8 have shown that the spatial patterns of the left- and right-hand motor imagery tasks for each individual subject are distinguishable. Additional calculations of the asymmetry of the power difference and sum from the symmetric channels (FC5-FC6, FC1-FC2, C3-C4, CP5-CP6, and P1-P2) would give a clear explanation for these results.  Figure 9 displays the asymmetries of the average power difference (a) and sum (b) of the mu and beta rhythms for all trials of the significant subjects. Outliers were excluded with criteria of ±5 standard deviation cutoff [56]. The asymmetries of all the symmetric channels were calculated using the following equation:

(17) $$\begin{array}{c} Asymmetry=\frac{{CH}_{L}-{CH}_{R}}{{CH}_{L}+{CH}_{R}}, \end{array}$$

where CH_*L*_ and CH_*R*_ indicate the symmetric channels of the left and right hemispheres, respectively. Therefore, when the power difference or sum from the left hemisphere is greater than the right, the resulting asymmetry will be positive and vice versa. Figures 9(a) and 9(b) use the power difference and sum of the mu and beta rhythms, respectively, from the symmetric channels.  Figure 9(a) shows a marked difference for the channels of the central area compared to Figure 9(b). Since the motor cortex responsible for all voluntary movements is located in the central region of the human brain, these asymmetry results can explain why the spatial pattern topographies of the left- and right-hand motor imagery tasks are prominently different in Figures 6 [Fig fig7]–8. In particular, in channels C3 and C4, the power difference asymmetries of the left- and right-hand tasks have different signs, meaning that the power difference of the mu and beta rhythms is greater in C3 during the left-hand task, whereas the power difference is greater in C4 during the right-hand task. Since this difference is only shown in Figure 9(a) and not in Figure 9(b), this could explain why the features containing the power difference between the mu and beta rhythms in the pseudocovariance matrix result in a better classification performance than those without the power difference information.

## 4. Discussion

### 4.1. Motor Cortex Channels

In this study, the channel data that covers all the brain regions was used. Therefore, an additional experiment using only the motor cortex channels was conducted, since the motor cortex is originally known to be responsible for the motor movement or imagery tasks [57]. The same data analysis methods from the original study were used. As shown in Table 7, the performances of all CSP algorithms were lower than those of Table 1. A reasonable explanation could be that supplementary information was kept in the occipital and the nearby parietal region, due to the perception of the visual stimulus guidance of the motor imagery tasks. Other studies using the positron emission tomography (PET) and the functional magnetic resonance imaging (fMRI) also showed that other areas including the parietal region, anterior cingulate gyrus, and the cerebellum were activated [58, 59]. Additional pairwise Student's *t*-tests to calculate *p*_1_ and *p*_2_ were conducted as shown in Table 8. This shows that SUTCCSP results in a higher performance even when using only motor cortex channels (*p*_1_ < 0.05 and *p*_2_ < 0.01, resp.).

### 4.2. Proposed Algorithm

In this study, MEMD was applied to the multichannel data, where channels were selected to cover all the brain regions. The nonlinear property of such real-world EEG data makes it difficult for the conventional frequency analysis methods to decompose the signals into the natural oscillations, such as Fourier transform based on the fixed sinusoidal functions. However, the data-driven MEMD method obtains the frequency components without any basis functions, and, thus, for the real-world physiological data, such as electromyogram (EMG) or electrocardiogram (ECG), MEMD has the potential to provide a highly accurate frequency analysis.

In addition, it is known that the left- and right-hand imagery is associated with bilateral desynchronization of mu rhythms, greater on the contralateral side, and the mu rhythm has prominent hemispheric asymmetry with the right-hand imagery, while the beta rhythm is more prominent with the left-hand imagery [20, 60]. Recent studies have shown the dissociation of mu and beta rhythms, which has not been identified in the previous studies [22]. Brinkman et al. showed an increase in the oscillatory power of the mu rhythm in the sensorimotor cortex ipsilateral to the tasks, whereas that of the beta rhythm decreased in the contralateral sensorimotor cortex simultaneously. The disassociation between mu and beta rhythms can explain why the complementary information of the power difference between mu and beta rhythms from the pseudocovariance could provide crucial information to classify the left- and right-hand motor imagery tasks. Also, the ipsilateral difference of the power difference of the left- and right-hand motor imagery EEG, shown in Figure 9, demonstrates that the complementary information of the power difference between mu and beta rhythms is an important feature for the classification of the left- and right-hand motor imagery tasks.

### 4.3. Processing Speed of CSP Algorithms

In order to calculate the processing speed of the system as in real-time applications, the computational complexity in terms of Big-*O* notation and the actual processing time of the three CSP algorithms were compared, since these algorithms were directly applied to the data segments of the test set data. The software used for calculation was MATLAB R2016a, since all codes were implemented using this software. The hardware specifications include a Windows 10 OS and an x64-based Intel(R) Core(TM) i7-6700HQ CPU (2.60 GHz) processor with 16.0 G of RAM.

When the computational complexity of the three CSP algorithms was calculated using Big-*O* notation, multiplication was calculated as *O*(*n* ^3^) since MATLAB uses the blocked-matrix multiplication method. The computational complexity of each algorithm was *O*(*n* ^4^) for all CSP algorithms. Although it may seem that there were no differences among the three methods, the actual processing time would be different, since the coefficients for SUTCCSP were much larger than those of CSP and CCSP due to more multiplication during processing a large number of features.

When calculating the actual computational time processed with the specifications mentioned above, approximately six trials were used for the test set since our study conducts a fivefold cross validation. The actual processing time of the three methods is shown in Table 9. Note that the unit of milliseconds (ms) would be of negligible difference among the different approaches for real-time processing.

### 4.4. Variation of Data Length

**Figure 10:**
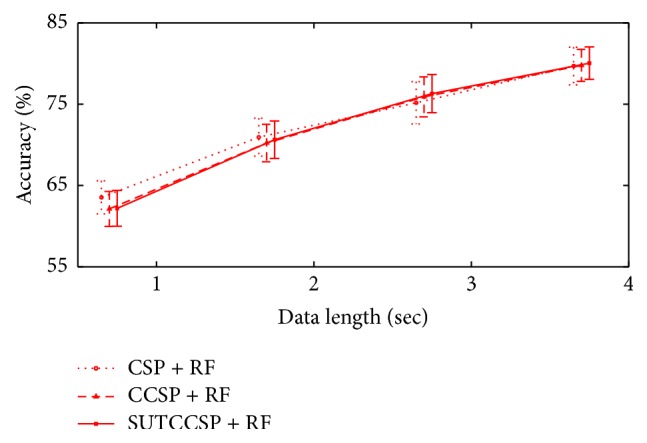
The errorbar of RF conducted with features from CSP, CCSP, and SUTCCSP when varying the data length. Note that the performance decreases as the data length reduces.

In order to search for performance differences when varying the data length, an additional experiment was conducted that shows the classification accuracies using data samples varying from 1 second (160 samples) to 4 seconds (640 samples). For benchmark testing, the best classification method, along with the significant subject pool from the CSP algorithms, was selected, since it yields the best results. Then, all experiments were processed from the preprocessing step with MEMD, feature extraction with CSP methods, and then classification. Results are displayed in Figure 10.

Figure 10 clearly shows that the data using a full data length of 640 samples increases the classification accuracy for all CSP algorithms. Therefore, it proves that the performance decreases when reducing the data length.

## 5. Conclusion

In this paper, we have used SUTCCSP to extract the different responses of mu and beta rhythms of EEG to the motor imagery tasks. Results showed improved classification performance using SUTCCSP with consideration of the power difference between mu and beta rhythms, compared to the original CSP algorithm. The functional disassociation between the mu and beta rhythms can explain the contribution of the supplementary information of the power difference to the motor imagery classification. Finally, our investigation of preprocessing and classification methods for the motor imagery EEG analysis has confirmed that MEMD and the random forest classifier are the optimal algorithms for this purpose.

## Figures and Tables

**Table 1:** Comparison of classification accuracies among different classification algorithms with significant subjects.

Classifiers	CSP (%)	CCSP (%)	SUTCCSP (%)
* RF *	* 78.04 ± 2.31 *	* 78.97 ± 2.11 *	* 80.05 ± 2.10*
LMT	77.16* ± *2.33	77.62* ± *1.97	77.75* ± *2.02
MT	76.89* ± *2.18	76.07* ± *2.04	76.02* ± *2.13
KNN	77.36* ± *2.35	77.49* ± *2.22	77.02* ± *2.28
LB	76.88* ± *2.17	75.98* ± *2.11	76.25* ± *2.25

**Table 2:** Comparison of classification accuracies among different classification algorithms with insignificant subjects.

Classifiers	CSP (%)	CCSP (%)	SUTCCSP (%)
RF	51.70 ± 0.78	50.87 ± 0.62	51.25 ± 0.71
LMT	49.72 ± 0.81	49.50 ± 0.79	50.59 ± 0.80
MT	49.71 ± 0.83	49.87 ± 0.78	50.70 ± 0.74
KNN	51.56 ± 0.57	51.03 ± 0.53	51.74 ± 0.55
LB	51.32 ± 0.63	50.79 ± 0.64	51.49 ± 0.67

**Table 3:** Student's *t*-test results, which compare classification accuracies of SUTCCSP with CSP (*p*_1_) and CCSP (*p*_2_).

Classifiers	*p*_1_	*p*_2_
*RF*	*0.0075*	*0.0037*
LMT	0.5055	0.7437
MT	0.2539	0.8798
KNN	0.6405	0.3620
LB	0.4736	0.5437

**Table 4:** Classification rates of significant subjects.

Preprocessing method	CSP (%)	CCSP (%)	SUTCCSP (%)
IIR	75.92 ± 2.04	76.06 ± 1.98	76.98 ± 1.90
*MEMD *	*78.04* ± *2.31*	*78.97* ± *2.11*	*80.05* ± *2.10*

**Table 5 tab5:** Student's *t*-test results for IIR and MEMD, which compare classification accuracies of SUTCCSP with CSP (*p*_1_) and CCSP (*p*_2_).

Preprocessing method	*p*_1_	*p*_2_
IIR	0.1115	0.0229
*MEMD *	* 0.0075 *	* 0.0037*

**Table 6:** Student's *t*-test results for MEMD versus IIR filter for all 105 subjects using the different CSP algorithms.

Feature extraction	*p* < 0.00001
CSP	0.000003
CCSP	0.0000005
SUTCCSP	0.000008

**Table 7:** Comparison of the classification accuracies of CSP algorithms using only motor cortex channels.

CSP (%)	CCSP (%)	SUTCCSP (%)
75.82 ± 2.24	76.62 ± 2.09	77.70 ± 2.09

**Table 8:** Student's *t*-test results, which compare classification accuracies of SUTCCSP with CSP (*p*_1_) and CCSP (*p*_2_) using only motor cortex channels.

*p*_1_	*p*_2_
0.0144	0.0090

**Table 9:** Actual processing time for CSP, CCSP, and SUTCCSP.

CSP (ms)	CCSP (ms)	SUTCCSP (ms)
11.227	9.894	30.272
